# High throughput, efficacious gene editing & genome surveillance in Chinese hamster ovary cells

**DOI:** 10.1371/journal.pone.0218653

**Published:** 2019-12-19

**Authors:** S. C. Huhn, Y. Ou, A. Kumar, R. Liu, Z. Du

**Affiliations:** Cell Line Development, Merck & Co., Inc., Kenilworth, NJ, United States of America; Lewis Katz School of Medicine at Temple University, UNITED STATES

## Abstract

Chinese hamster ovary (CHO) cells are a common tool utilized in bioproduction and directed genome engineering of CHO cells is of great interest to enhance recombinant cell lines. Until recently, this focus has been challenged by a lack of efficacious, high throughput, and low-cost gene editing modalities and screening methods. In this work, we demonstrate an improved method for gene editing in CHO cells using CRISPR RNPs and characterize the endpoints of Cas9 and ZFN mediated genetic engineering. Furthermore, we validate sequence decomposition as a cost effective, rapid, and accurate method for assessing mutants and eliminating non-clonal CHO populations using only capillary sequencing.

## Introduction

Chinese hamster ovary cells, or CHO, are the lynchpin of modern biotherapeutic manufacturing and serve as the most ubiquitous mammalian expression platform [1, 2]. Optimizing the CHO expression system is of exceptional interest to improve the output, quality, and stability of biologics. Historically, the development of CHO hosts has been largely been the result of brute-force phenotypic screening and this has yielded many of the lineages utilized by the biopharmaceutical industry today [3]. Currently, major advances in gene editing technology have driven a rapid expansion of directed host cell line improvement efforts [4–8]. This boon has resulted in a demand for host engineering timelines to meet or exceed that of typical biotherapeutic pipeline projects.

Genetic engineering is typically accomplished through artificially engineered proteins, such as Zinc finger nucleases (ZFNs), transcription activator-like effector nucleases (TALENs), or naturally occurring RNA-guided nucleases (RGENs), such as CRISPR/Cas9 [9, 10]. Whereas ZFNs and TALENs are engineered proteins that consist of DNA binding domains fused with a nuclease, CRISPR/Cas9 represents a class of naturally occurring bacterial endonuclease that can be repurposed for selective mammalian gene targeting. Practically speaking, the major obstacle of utilizing artificial constructs, such as ZFNs or TALENs, is the degree of protein engineering required to generate a stable and efficient molecule [10, 11]. ZFN or TALEN design is laborious, requiring hundreds of design iterations which usually result in efficiencies ranging from 10–50% [10, 11]. In contrast, CRISPR Cas9 mediated genome editing represents a revolution in genetic engineering due to its exceptional flexibility and ease of use, requiring only a twenty nucleotide sequence known as a gRNA (guide RNA), followed by a three nucleotide motif within the genome (protospacer adjacent motif or PAM), to target any genomic locus of interest [9]. CRISPR efficiencies are generally exceptional (upwards of 90% has been reported without selection) and typically only a handful of gRNAs need to be assayed for any given locus [9]. Therefore, CRISPR is especially attractive when speed and throughput are paramount.

The emergence of flexible gene editing technologies has also mandated a demand for accurate, fast, and cost-effective metrics to assess gene editing efficacy. Historically, quantification of gene editing has been reliant on gel-based endonuclease assays, which function on the principal that the frequency of mutations in a sample is proportional to the amount of endonuclease-driven DNA cleavage [12]. This assay experiences several drawbacks: T7 is insensitive to small changes within mutant alleles (such as single nucleotide polymorphisms), quantification is difficult (relying on gel-based densitometry or other equipment), and the nature of the mutations within the sample cannot be characterized [12]. Recently, computational approaches capable of deconvoluting sanger traces into individual parts such as TIDE, INDIGO, and ICE have showed exceptional promise [13, 14]. Utilizing only a PCR reaction and a capillary sequencer, these algorithms estimate both efficiency and complexity through combinatorial alignment of wild-type and gene edited sanger trace files. While next generation sequencing platforms represent the highest standard for gene editing readout, cost, speed, and computation labor involved in these systems tends to be prohibitive. Therefore, deconvolution algorithms are especially attractive in biopharmaceutic production where cost-effective, high throughput, and fast genome screening is required.

In the context of CHO cell line development, stringent timelines mandate an ever-increasing need for more efficient assays and rapid testing methods. Therefore, we sought to assess the feasibility and improve methodologies of genome editing and screening. In this work, we utilize the glutamine synthetase (GS) gene, a popular choice in CHO genome editing, as a surrogate locus to characterize CRISPR and ZFN gene editing modalities and screening methods. We demonstrate critical advantages of CRISPR RNPs and show how deconvolution algorithms can be utilized in place of cost-prohibitive and expensive NGS platforms to analyze editing outcomes and selectively eliminate nonclonal and wildtype cells.

## Material & methods

### CRISPR complex generation

CRISPR guide RNAs (gRNAs) were designed according to procedures already outlined [15]. CRISPR RNAs (crRNAs) were synthesized by IDT and were combined at a 1:1 molar ratio with ATTO-550 labeled (Excitation: 560nm, Emission: 575nm) trans-activating crRNA (tracrRNA; IDT, Cat: 1075928) in 30 mM HEPES, pH 7.5; 100 mM Potassium Acetate. The RNAs were then heated to 95ºC for 5 minutes on a heatblock and allowed to reach room temperature. The duplexed gRNAs were complexed with purified Cas9 protein (NEB, Cat: M0646) at room temperature for 20 minutes before transfection.

### ZFN mRNA generation

ZFN mRNAs were prepared from two plasmids (ZFNGSA9075 and ZFNGSB9372, Sigma) expressing a pair of Zinc Finger nucleases targeting the CHO glutamine synthetase gene. The two plasmids were first linearized by XbaI, and then subjected to *In Vitro* transcription using the HiScribe T7 ARCA mRNA Kit (NEB, cat: E2060S). The two paired-ZFN mRNAs were purified using the MegaClear Kit (Thermo, Cat: AM1908), combined, and then aliquoted in RNase-free tubes. In the case of OLFR613, custom plasmids were obtained from Sigma Aldrich (Saint Louis, MO)

### Generation, propagation, and screening of KO cells

Merck suspension CHO hosts, derived from CHOK-1, were grown in CD-CHO medium (Gibco, Cat: 10743029) containing 1×HT Supplement (Gibco, Cat: 11067030) and L-Glutamine (Gibco, Cat: 25030081). CHO cells were continuously cultured every three days in shaking incubator (Kuhner) at 37°C, 5% CO^2^ with or without L-glutamine.

CHO cells were transfected with either CRISPR complexes or ZFN mRNA by electroporation according to manufacturer’s protocol (Thermo Fisher Scientific). After electroporation, the transfected cells were transferred into well plates in a static incubator. Cell viability and count was assessed daily using a ViCell XR (Beckman Coulter). Three days following transfection 1 ×10^6^ cells were lysed and extracted for gDNA using the GenElute kit (Sigma, Cat: G1N70) according to the manufacturer’s instructions.

For cloning, the bulk pools were cloned into 96-well plates through limiting dilution or single cell sorting by using FACS Fusion sorter (BD). After approximately 10–14 days, each colony was screened for GS gene disruption according the manufacturer’s instructions (Lucigen, Cat: QE0905). gDNA extract was then transferred to a PCR reaction plate with the mastermix and thermocycler conditions below. The PCR reaction was then submitted for Sanger sequencing following an enzymatic cleanup. Alternatively, genomic DNA was isolated as described in the bulk pool stage.

### PCR

PCR reactions in this study utilized AccuPrime Pfx DNA Polymerase (Invitrogen, Cat: 12344024) and an ABI Veriti thermocycler. The reactions proceeded identically to the manufacturer’s recommendations, except that 100 ul of reaction volume was used per reaction with 100 ng of input gDNA, using an annealing temperature of 68C. Each reaction did not exceed 30 cycles. The primer pairs identified in Table 1 were utilized:

**Table 1:** Primer pairs utilized in this study.

	Sequence (5' → 3')
GS-F	GACAAACACGAAGAGCATGGCA
GS-R	TGGGCAGTAGTTCTACCAAGGC
OLF613F	AGACAGGCATCCAGACCAAC
OLF613R	AGTAATGCAATCGCTGGGTGA

### T7 endonuclease assay

PCR products were purified (Qiagen, Cat: 28104) and eluted in molecular biology grade water and were then adjusted to 1X NEB Buffer 2.1 (NEB, Cat: B7202S). Products were then boiled for 10 minutes, allowed to cool to room temperature, divided in two, and treated with 1.5ul of T7 Endonuclease (NEB, Cat: M0302S) or water. The reaction proceeded for 1hr, until it was deactivated by adjusting the mixture with 1X Purple loading dye (NEB, Cat: B7024S). The reaction was then run at 120V for 65 minutes on a 1.5% agarose gel. The band intensities were quantified by densitometry using image-j according to previously published procedures [16]. For fragment analysis we utilized the method by Ran et al [16]. Results represent two to three independent experiments and error bars reflect standard deviation from the mean.

### Sanger sequence decomposition

Indefinite mutations induced by gene editing were assessed by two independent decomposition algorithms (TIDE or ICE). Briefly, mutant and wildtype cells were lysed, and gDNA was amplified utilizing primer sets described above. The PCR products were purified and subjected to Sanger sequencing.

For TIDE, trace files were aligned to the wildtype reference using an estimated Indel size length of 1–30, along with a 115–500 bp decomposition window and a 100bp alignment window. We utilized 5’ GCCATACCAACTTTAGCACC 3’ as the expected cut-site (gRNA) for the ZFN mRNA. Traces were analyzed with a p<0.0001 cutoff value and output files with an R^2^ value of less than 0.95 were eliminated from further analysis. Results represent two to three independent experiments and error bars reflect standard deviation from the mean.

For ICE, the algorithm was obtained from GitHub and deployed according to the manufacturer (https://github.com/synthego-open/ice) using the same estimated cut-site and statistical parameters.

### Next generation sequencing

Amplicons were assayed using an Invitrogen Quant-iT dsDNA (Thermo, Cat Q33120) assay followed by electrophoresis to determine DNA concentration and quality. Samples were then used to generate libraries using the Illumina TruSeq Nano DNA kit (Illumina, Cat: 20015964). The concentration and size range of the generated libraries was then determined using the Quanti-iT dsDNA Assay kit, and libraries were sequenced using the Illumina MiSeq platform with read length of 2x150bp. 4.5Gb of sequencing data was generated per DNA sample.

Prior to data analysis, samples were demultiplexed using bcl2fastq-v.1.8.4, and adapter sequences were trimmed using trim_galor_v0.3.3., BBDuk (http://jgi.doe.gov/data-and-tools/bb-tools/) was utilized for an additional cleanup step. Briefly, right end adapters with a 27-Kmer length were trimmed with a maximum substitution setting of 1. Low quality reads were trimmed from both ends with a minimum quality of 30 and short reads less than 70 base pairs were discarded. Duplicates were removed, and adapters were trimmed based on paired read overhangs with a minimum overlap of 24.

For CRISPRESSO, the amplicon sequence was utilized as a reference and reads with a phred quality of less than 30 were discarded [17].

### Fluorescent microscopy and flow cytometry

Twenty-four hours after CRISPR transfection, ATTO-550 (Excitation: 560nm, Emission: 575nm) transfected cells (0.1x10^6^) were aliquoted in a 48 well plate and imaged by microscopy. Cells were analyzed on a Nikon Eclipse Ti-S (Nikon Instruments) with a Texas Red (563 nm) filter set. Utilizing the Nikon Elements software package, the mean fluorescent intensity was calculated by measuring the fluorescent intensity from each cell in a constant, predefined area. Background fluorescence was subtracted from each image and greater than forty cells were analyzed per condition. For flow cytometry, 1 x 10^6^ control cells or cells transfected with ATTO-550 labeled tracrRNA were analyzed on a BD FACS Fusion with a Texas red laser (595 nm) until 10,000 events were captured. For enrichment, the top 20% of ATTO-550 labeled cells were bulk sorted, lysed, and subjected to sequencing as above, or allowed to recover 48 hours before cloning by limiting dilution.

### Generation and selection of recombinant cells

Suspension CHO cells were transfected with GS expression vector DNA utilizing electroporation. The transfected cells were then selected with CD-CHO media without glutamine, supplemented with 0–50 uM methionine sulfoximine (MSX). After continuous subculture, stable pools were cryopreserved and evaluated by fed-batch production assay.

### Fed batch production

Cells were seeded in in-house production media and glucose & lactate levels were measured daily using the RANDOX RX imola chemistry analyzer (Crumlin, UK). Cell density and viability were measured using a Beckman Coulter ViCELL cell counter (Beckman Coulter, Indianapolis, IN). mAb production levels were determined by Protein-A UPLC.

### Statistical analysis

In all assays Graphpad Prism was utilized to calculate scores (using ANOVA or student’s T-test) to judge statistical significance.

## Results

**Fig 1:**
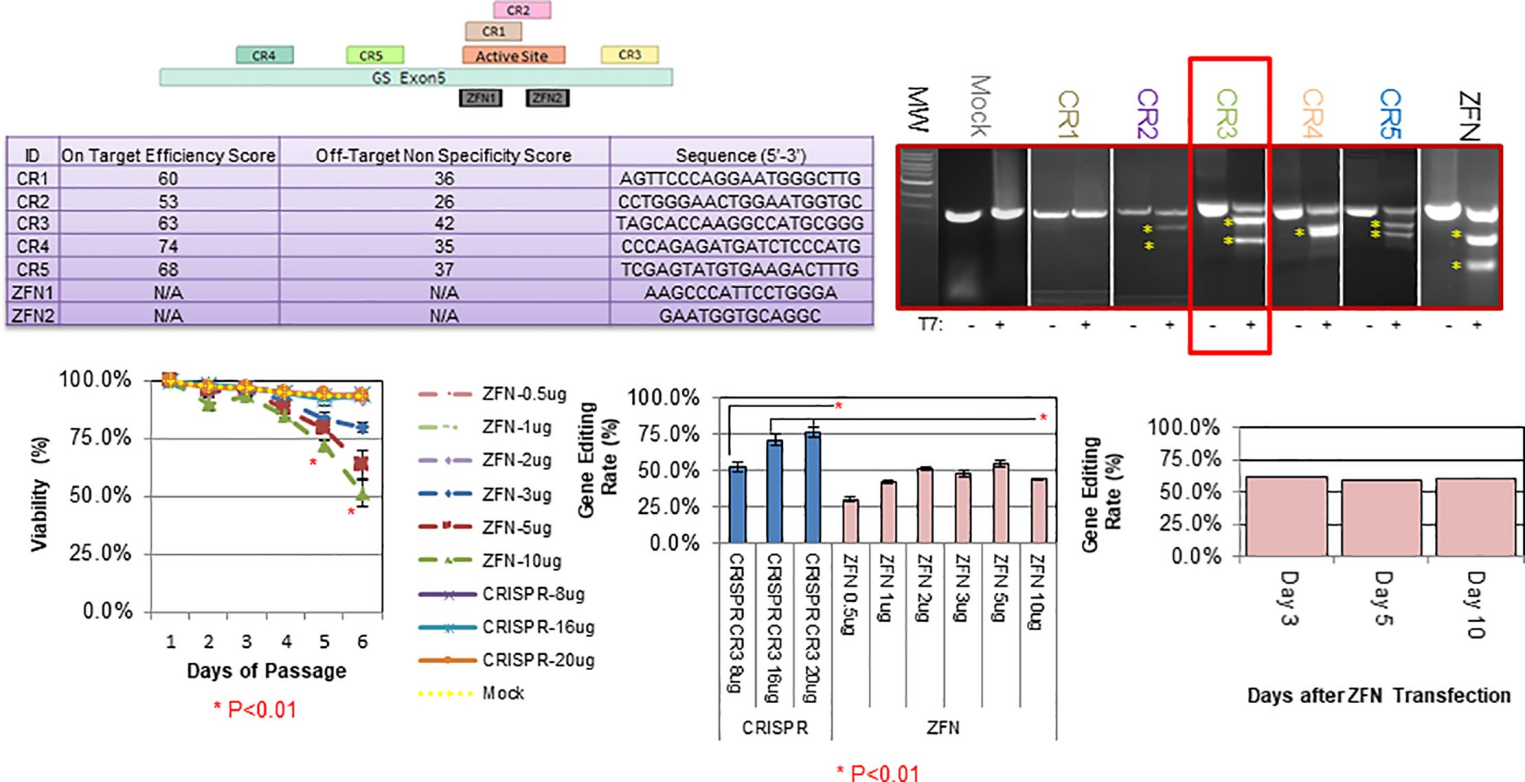
Comparison of ZFN mRNA vs. CRISPR RNP at the glutamine synthetase locus. Five gRNAs were designed to target GS exon 5. The active site is designated in light red, while the gRNA binding sites are labeled CR1-5 **(top left panel)**. ZFN binding sites from Fan et al. are indicated as the grey ZFN1 and ZFN2 boxes. Doench scores demonstrating the predicted on-target (cleavage) efficiency as well as the predicted off-target (cleavage of non-target sites) are predicted in silico within the table (**left hand table**). Cells were then transfected with CRISPR RNPs 1 through 5, and gDNA was PCR amplified & subjected to a T7 endonuclease assay, with or without addition of nuclease (shown by a + or—; **top right hand panel**). Yellow asterisks indicate cleavage products, and successful editing. The most efficacious gRNA, CR3, is boxed in red. CHO cells were then transfected with the indicated amounts of Zinc Finger mRNA or Cas9 CR-3 RNP. Cells were assessed daily for viability **(bottom middle panel, D5&6 ZFN 3-10ug P<0.01 vs mock).** Two days after transfection, equal aliquots of cells from each were lysed and the percentage of mutant alleles were quantified **(middle panel, CR3 16ug or CR3 20ug P<0.01 vs all ZFN conditions).** Significance using student’s T-test is demonstrated by a red asterisk. To ensure temporal effects did not result in reduced efficacy of the ZFN mRNA vs CRISPR RNP, ZFN transfected cells were continually passaged and then lysed and analyzed five and ten days after the initial transfection **(bottom right hand panel)**.

The advent of directed genome editing has resulted in a demand for the genetic manipulation of CHO hosts under an accelerated timeline. Established methods demonstrated a yield of 2–5% biallelically modified clones [18] and we therefore first sought to identify approaches to improve the efficiency of our pipeline. We directed our attention to CRISPR RNPs as they offer several critical advantages in the context of biotherapeutics and mitigate concerns regarding use of animal components, integration into the host, and off-target effects [19, 20]. We developed an optimized protocol (S1 Fig and Material and Methods), for efficient delivery of CRISPR RNP complexes into suspension CHO cells using fluorescently tagged synthetic tracrRNA, which resulted in >90% transfection efficiency and >80% efficacy without selection, and which could be further improved by FACs. Next, to better understand gene editing outcomes and improve on established methodologies, we contrasted CRISPR RNPs and ZFN mRNAs at the glutamine synthetase gene, an attractive target in CHO bioproduction which has been well characterized [17]. We directly compared five independent CRISPR gRNAs alongside the canonical ZFN mRNAs utilized to generate GS-null cell lineages (Fig 1 upper panels) [17]. The ZFN mRNA modality resulted in toxicity at high concentrations (Fig 1, ZFNs 3ug, 5ug, and 10ug from D3-D6; P<0.01 vs Mock) and was less efficient than the most efficacious CRISPR RNP (Fig 1, middle panel, ZFNs vs CRISPR CR3 16 & 20ug, p<0.05), suggesting multiple advantages for the use of RNPs over long ZFN mRNAs.

**Fig 2:**
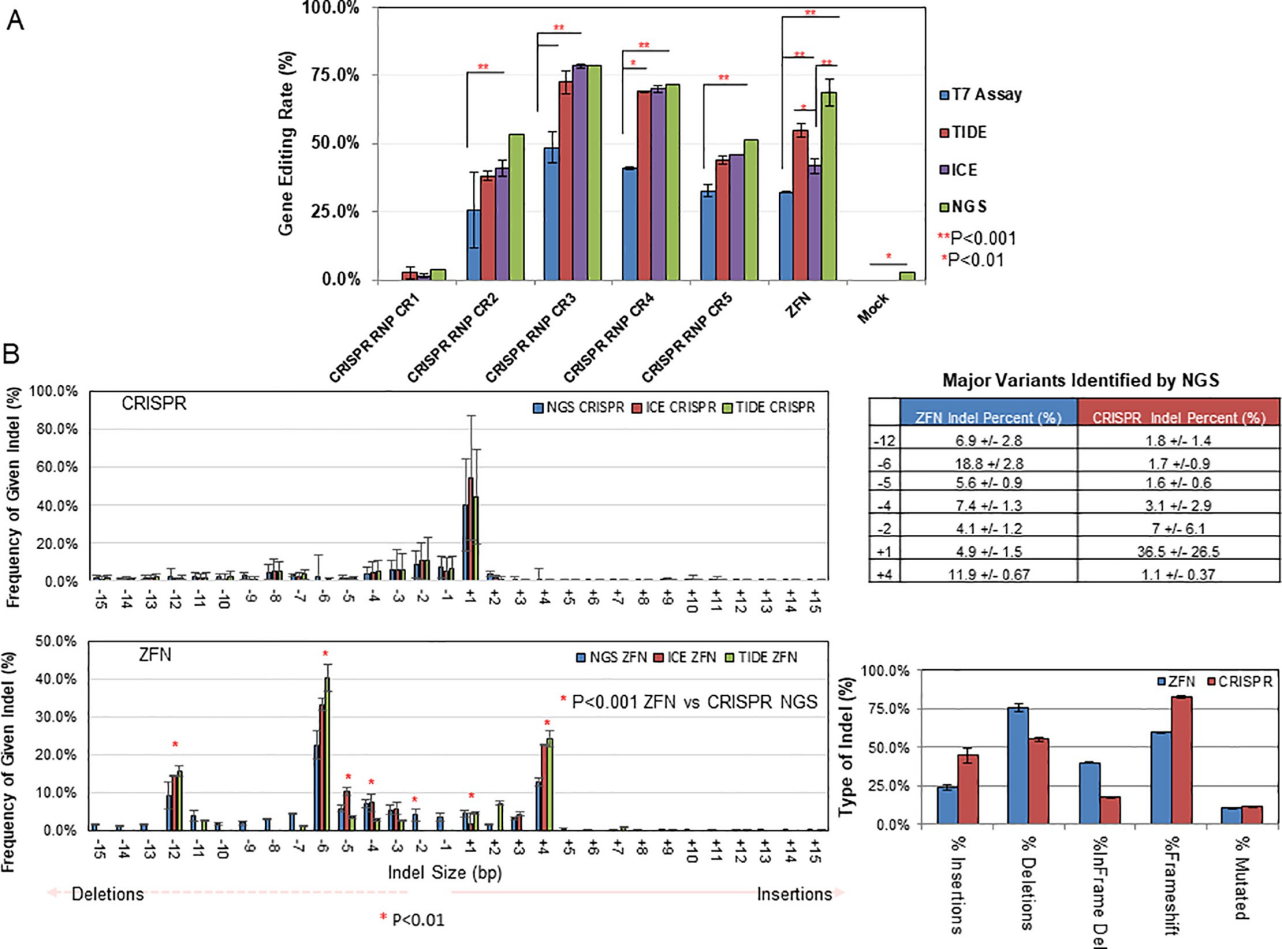
The ZFN vs. CRISPR footprints are unique and are detectable using sequence decomposition. **(A)** Gene edited CRISPR or ZFN pools of across glutamine synthetase exon 5 were subjected to lysis and PCR 72 hours after treatment. Aliquots of each amplicon was then quantified using TIDE, ICE, NGS, or by densitometry (**top panel)**. Statistical comparison between are shown using a student’s T-test between conditions, with the indicated symbols representing when significance was reached. **(B)** CRISPR (top panel) and ZFN (bottom panel) transfected cells were assessed by NGS (blue histograms bars) ICE, (red histograms bars) or TIDE (green histograms bars). The results of 4 independent CRISPR edited pools were pooled and the mutation events were directly compared to ZFN mutations. The left-hand side of each graph represents the spectrum of internal deletion sizes in each given cell population, ranging to 1 to 15bp in size, while the right-hand size represents insertions ranging from 1bp to 15bp in size. The read frequency represents the percentage of each allele among all mutant reads. The variants demonstrating a significance change via two-way ANOVA between pools are indicated with a red asterisk and are further shown in the **right-hand table**. The type of mutation observed in each population of CRISPR or ZNF transfected cell type is depicted in the **bottom right hand graph**.

Improved readout efficiency is also needed for commercial development if the promise of high-throughput gene editing is to be realized. Thus, we also sought to improve throughput in the context of accurate and fast genome screening. Four independent methods were evaluated: the T7 endonuclease assay, sanger trace decomposition (using TIDE or ICE), or targeted next-generation sequencing (NGS) combined with bioinformatics [16]. Genomic DNA from each of the RNP or ZFN treated pools was isolated and subjected to PCR and a portion was then subjected to each assay. NGS of the PCR products delivered >10,000 reads (phred score of >35) and therefore was chosen as the gold standard of evaluation, as discussed previously [12]. We observed that treatment of the PCR products with T7 endonuclease followed by gel-quantification was the least robust method of assessment, deviating from the NGS results by as much as two-fold [12]. NGS and both sequence decomposition methods reported significantly higher editing rates than the T7 assay (Fig 2A, compare blue bars vs all colors), but there was a tendency for decomposition to underestimate editing as compared to NGS as the indel size became larger (Fig 2B, ZFN indel spectrum). This was probably due to the relatively short alignment window following the cleavage site in the PCR amplicon. These results suggest that sequence decomposition can reproduce next-generation data in the context of gene editing efficiency. Next, we asked the question if decomposition analysis could correctly characterize the mixture of mutations found in these pools. To answer this, we combined the editing outcomes from the gRNAs 2–5 edited pools and contrasted them to the Indels generated by the ZFN pair within the same ~500bp region. CRISPR mediated editing resulted in shorter Indels as compared to the ZFN pair, and these tended to result in more frameshift mutations (Fig 2B, top panel). These results were well recapitulated by the two independent decomposition methods, though both tended to over-report the most common mutations in each (Fig 2, compare green and red histogram bars vs. blue) and underreport those less obvious. Furthermore, a comparable spectrum of Indels was observed utilizing different gRNA/ZFN sequences at an independent locus within the CHO host (S2 Fig) confirming that this phenomenon was not locus specific. These data demonstrate that unique outcomes resulting from different gene editing technologies occur and can be determined by sequencing decomposition, especially complex populations.

**Fig 3:**
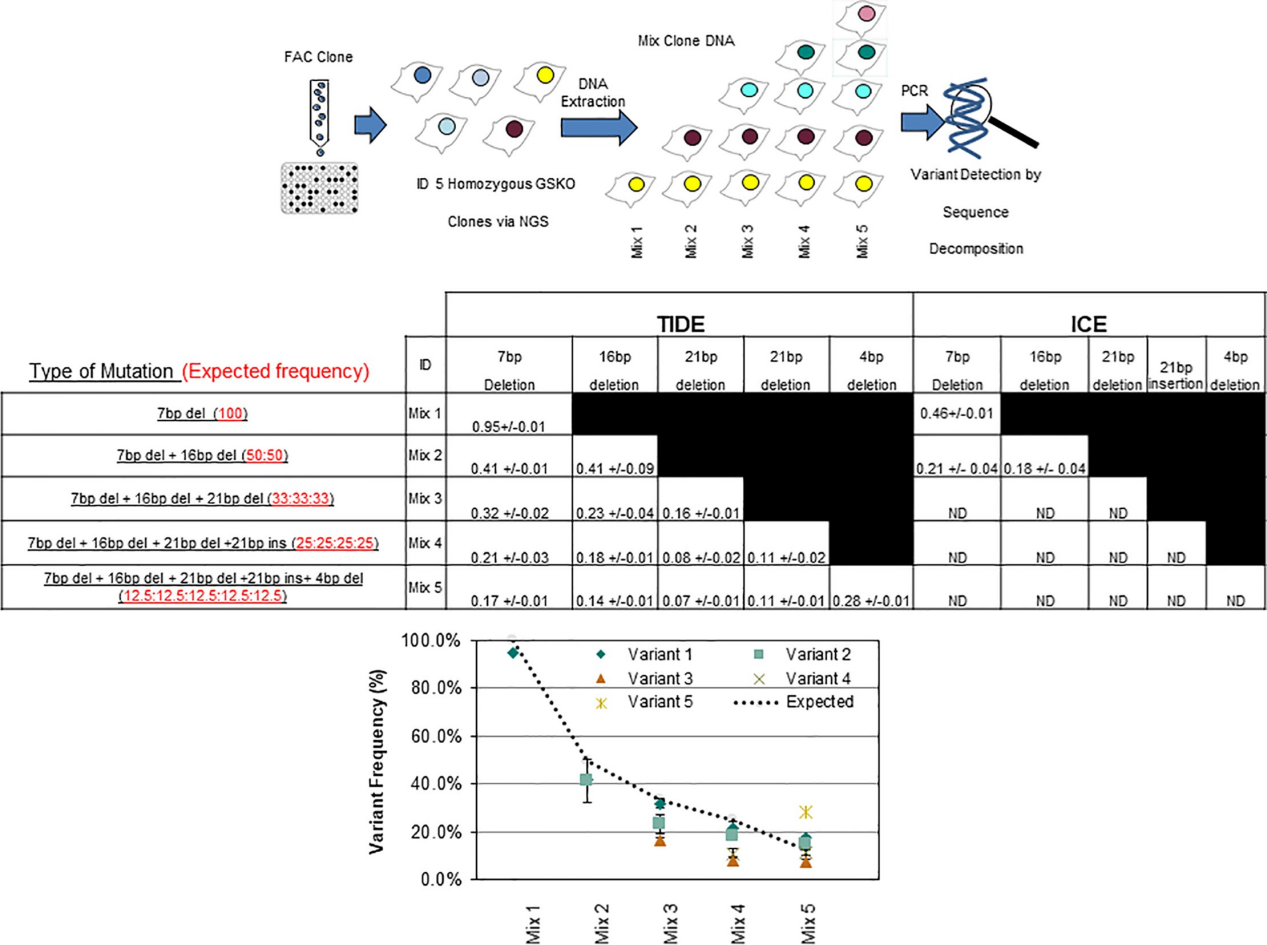
Sequence decomposition can successfully detect variant frequencies samples derived from multiple clones. The gDNA from five FACs sorted, NGS-verified clones containing homozygous mutations was mixed and subjected to PCR followed by TIDE or ICE **(top illustration).** The expected ratios and the identity of each variant are shown the middle table in the **far left-hand side**. Variant frequency observed from each assay is reported in the **center and far right panels of the middle table**. TIDE analysis was then plotted as a function of deviations the baseline expectation and is shown in **the bottom panel graph**. ICE analysis was omitted for plotting purposes as it could not distinguish >2 variants (right side of table). ND = Not detected.

We next focused on testing the sensitivity limits of decomposition algorithms in the context of individual cell clones. To achieve this, genomic DNA from five FACs sorted homozygous mutant clones (previously identified by NGS) was isolated, normalized, and combined in stepwise manner as indicated by the illustration in Fig 3. This mixture was then PCR amplified and analyzed by TIDE or ICE to detect the frequencies of each Indel. We observed that all the variants in each mixture were identified by TIDE, though TIDE did not always match the expected frequencies, especially when the sample became more complex (Fig 3, graph and left-hand side of table, compare Mix-2 to Mix-5). Alternatively, ICE was able to identify the dominant variant in all homozygous clones, but incorrectly reported the presence of wild type sequence, which compromised the true frequencies of mutant variants (Fig 3, right-hand table). Additionally, when more than three genotypes were assayed, the ICE the algorithm was incapable of identifying the original variants (reported as ND in the table). Therefore, we exclusively utilized TIDE to move forward in our analysis.

**Fig 4:**
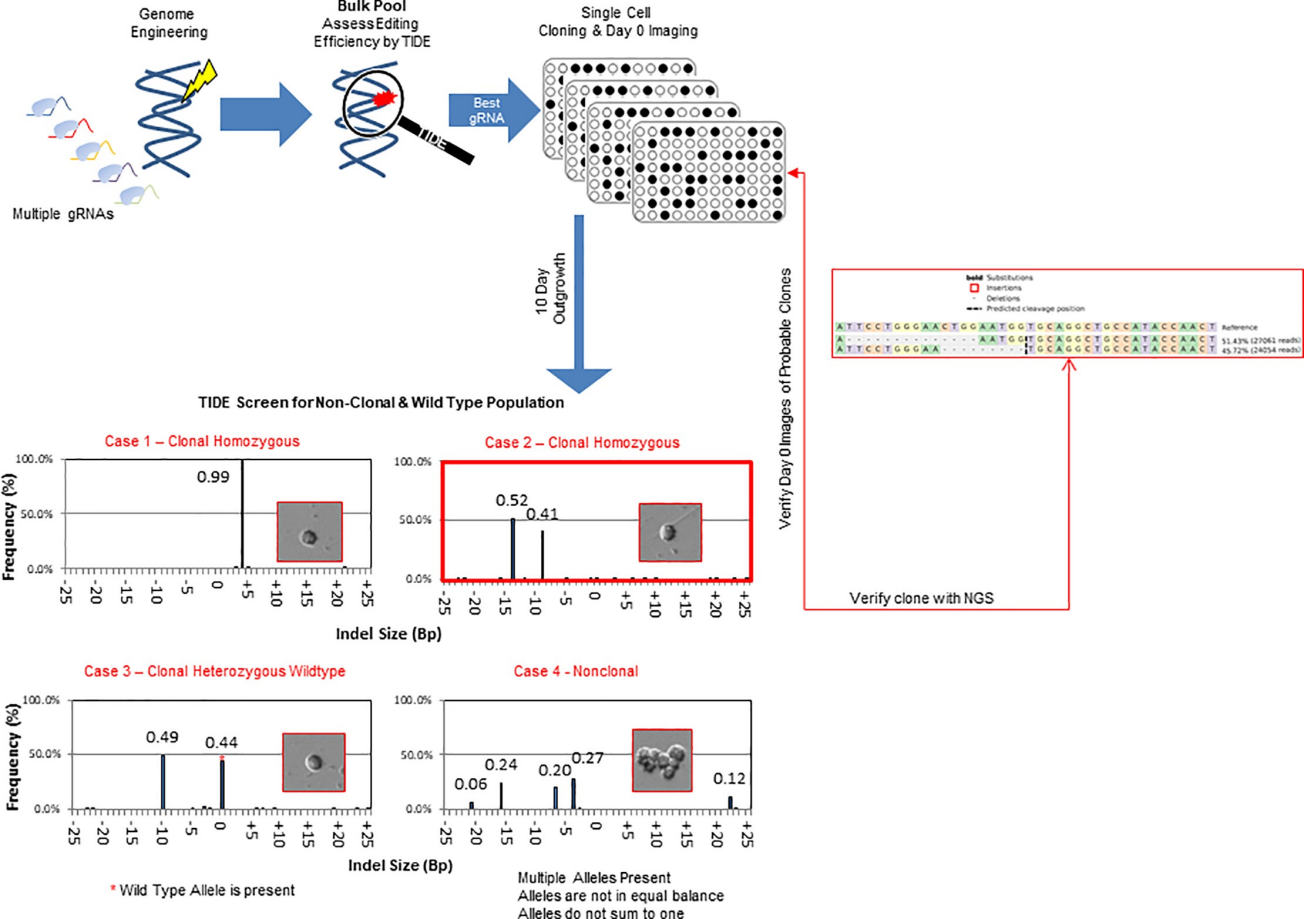
TIDE is an effective method for simultaneously identifying non-clonal and wild type populations during cell line development. Flow chart for high-throughput CHO clone screening: cells are first screened for gene editing efficacy at the bulk pool level using sequence decomposition to identify the most promising gRNA or molecule and then cloned **(top left-hand graphics)**. Clones are imaged at day zero; after 10–14 days of outgrowth a fraction of the well is aliquoted, lysed, and assayed via TIDE. Wells containing wild type Indel variants **(bottom left histogram and image)** and Indel variants in a greater frequency than three **(bottom right histogram and image)** are eliminated from further consideration, while homozygous or compound heterozygous mutant clones are retained **(top left and right histograms and images)**. The frequency of alleles and their associated day zero images are juxtaposed next to the indicated TIDE variant frequencies. Wild type alleles are indicated by a red asterisk. Clones retained from the filtering step are later confirmed by day zero imaging and eventually next-generation sequencing **(far right alignment).** Results of the same sample assayed by NGS, TIDE, and imaging are boxed in red.

Clonality plays a critical role in the context of biotherapeutic production as clonal lineages are thought to offer a more consistent product quality profile [21]. We reasoned that in the context of gene editing, significant deviations from homozygous or compound heterozygous frequencies, (i.e. a 100% or 50:50 mix of internal deletions) could be utilized as a crude, but rapid metric for distinguishing if an edited population originated from more than one progenitor cell. Using this strategy, we chose to employ TIDE as a filter for both estimating clonality and genetic functionality in the early stages of cell screening (Fig 4, see flow chart). Cells with variants >2 were presumed to originate from more than one progenitor and were discarded (bottom right hand panel), together with cells demonstrating wild-type variants (bottom left hand panel). Using this procedure, we were able to enrich for populations with either homozygous (top left-hand panel) or compound homozygous mutations. To safeguard against wells containing multiple cells with identical indels, we later utilized imaging to confirm clonality as a late stage filter. Following this filtering step, a reduced number of clones needed to be verified by tedious single cell imaging and/or next generation sequencing, saving on both cost and time.

**Fig 5:**
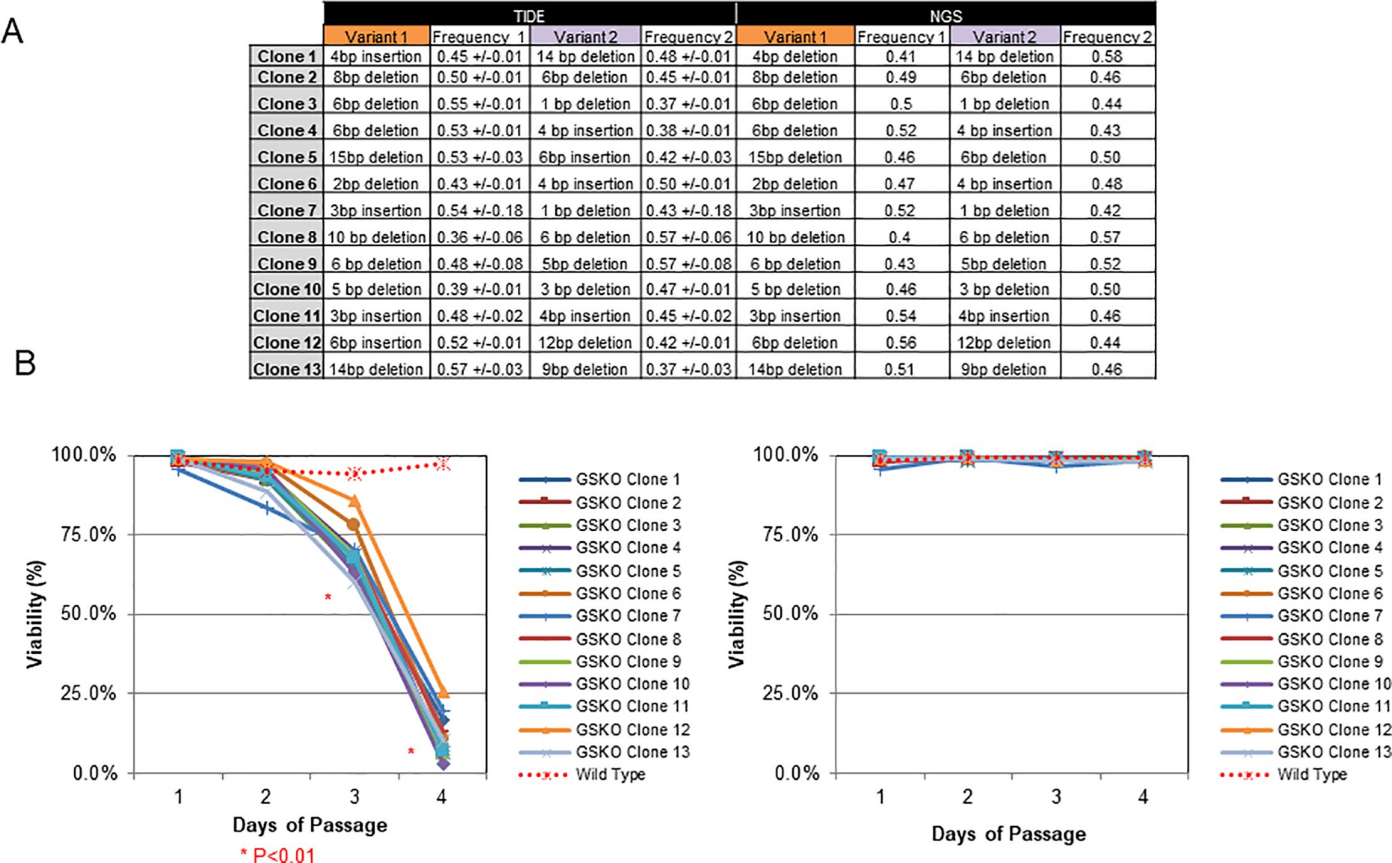
TIDE can identify mutant clones with accuracy close to NGS. **(A)** Thirteen compound heterozygous clones were identified by TIDE, confirmed by, imaging and the frequency and the standard deviation of each allele was measured by two independent TIDE assays **(white frequency columns, left hand of the table).** The frequency and identity of each variant was then quantified by NGS (**white frequency columns, right hand of the table**). **(B)** Clones in panel **A** were grown without **(left hand panel)** or with glutamine **(right hand panel).** The viability was recorded after every passage and plotted. The red asterisk indicates data points (all day 3 and 4 mutants, student’s t-test vs control) with a significantly changed viability from the control sample.

**Fig 6:**
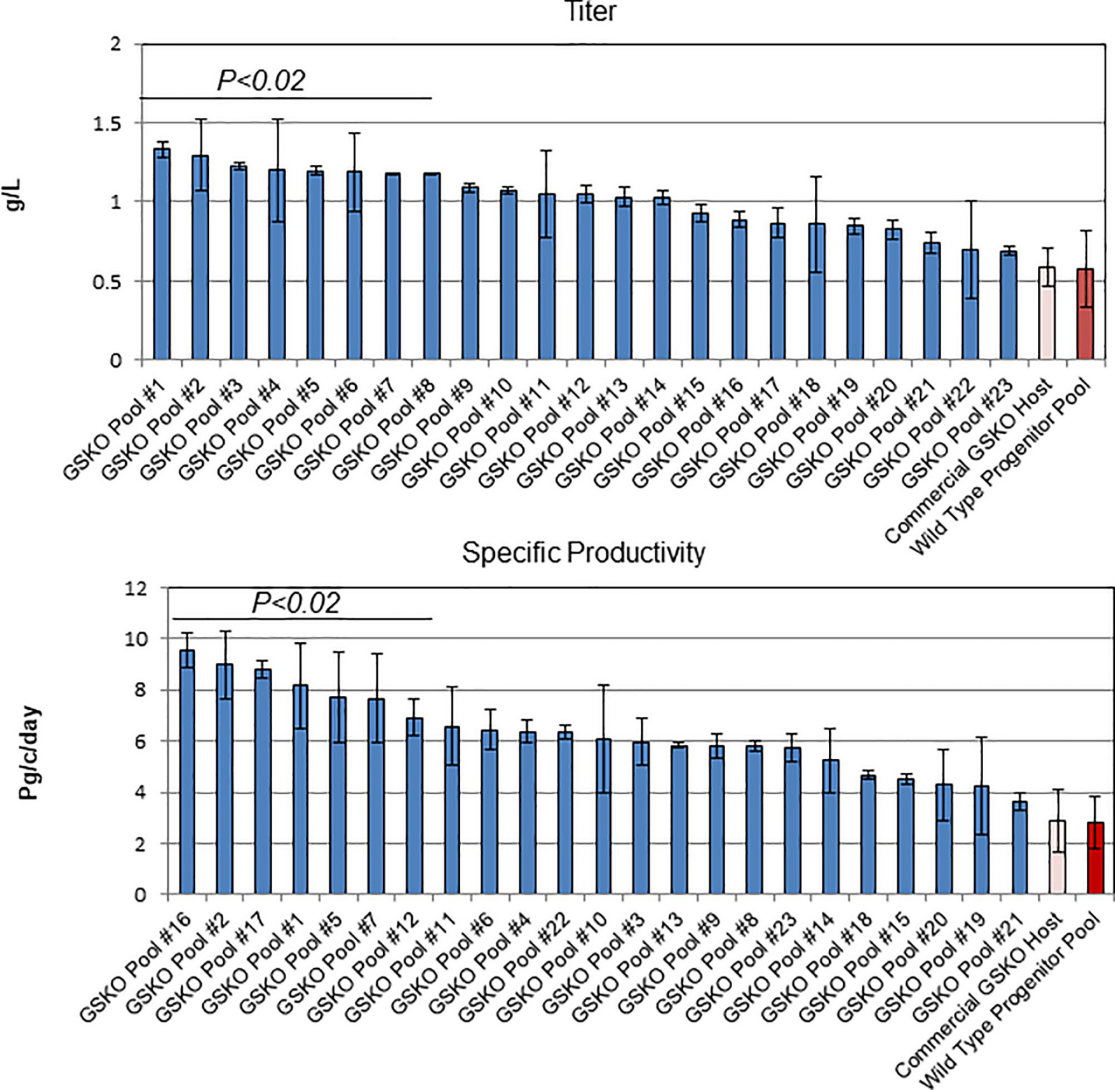
Generation of recombinant cell lines from GS null hosts. Twenty-three GS null recombinant pools, one wild type control, and one commercial GSKO cell line were transfected with recombinant mAb plasmid containing a GS expression cassette. Following selection, bulk pools were entered a 14-day fed batch. After 11 days, the cell titer **(top panel)** and specific productivity **(bottom panel)** were assessed. The samples which showed a statistically significant increase (P<0.02, one-way ANOVA vs. wild type pool) from the wild type pool are depicted under the black line.

As a proof of concept, we cloned edited pools by limiting dilution, and challenged TIDE to identify only compound heterozygous samples. We observed that TIDE was close (within +/- 6.7 on average) in quantifying the mutated variants within each heterozygous clone as compared to NGS (Fig 5). In addition, TIDE was successful in eliminating both non-clonal populations during cloning and heterozygous clones with a wildtype allele (Fig 4, bottom panels). Furthermore, when these clones were challenged with glutamine deprivation, we observed a loss of culture viability, demonstrating that TIDE could correctly identify null clones (Fig 5). Last, to determine if the identified clones were functionally useful, we transfected mutants with antibody expressing plasmids with exogenous GS, performed selection, and transferred to production media for a 14-day fed batch culture. Cell-free culture supernatants were harvested on day 11 and were analyzed by Protein-A chromatography. As shown in Fig 6, all clones assayed demonstrated superior productivity, titer, and product quality as compared to the wild type control (clones falling under the solid line reaching significance).

## Discussion

In this work, we characterized resultant mutations from both ZFN and CRISPR gene editing modalities in CHO cells and compared metrics for gene editing in a high throughput setting. We demonstrated a highly effective method for transfection of suspension CHO cells with CRISPR RNP payloads and establish that fundamental differences occur in the editing outcomes of CRISPR gRNAs vs ZFN pairs. The differences between these modalities have been highlighted in the past [22], but to our knowledge an exact comparison within proximal binding sites has not been well described. We suggest that this finding is likely the result of spacing between the paired Zinc Finger DNA binding domains (S2 Fig), which can result in enrichment of specific indels at that cleavage site [23, 24]).

We took advantage of the differential Indel spectrums induced by both ZFN and CRISPR to validate metrics for assess gene editing and show how deconvolution-based methods can be utilized in the context of cell line development. Most promisingly, we show how variant identification can be exploited as a tool to simultaneously eliminate non-clonal and wild type cells. We conclude that the step-wise high throughput clone generation and screening approach described in this report could be applied to other similar gene-editing efforts to further improve and optimize CHO host cell lines for the manufacturing of therapeutic biologics.

## Supporting information

S1 Fig Optimization of CRISPR RNP delivery in CHO cells. **(A)** CHO cells were transfected with 25 picomoles of Cas9 nuclease (4ug) while titrating the amount of gRNA **(left hand panel)**. Cells were then transfected with the indicated amount of Cas9 nuclease while maintaining a fixed 4:3 nuclease:gRNA mass ratio **(or 1:1:1.7 moles; right hand panel)**. The percent gene editing efficiency in each condition was then quantified and graphed. The red boxed bars in each graph demonstrates significance (p value indicated) vs other samples but not each other. **(B)** CHO cells were then transfected with the indicated amount of fluorescent RNP and imaged using fluorescent microscopy **(center image panels).** The mean fluorescent intensity of the cell population was quantified by microscopy **(far right-hand panel)** and percentage of transfected cells determined by flow-cytometry is demonstrated under each image. The representative fluorescent shift between the control and transfected cells, determined by flow cytometry is represented in the **bottom hand panels** and the top 20% expressers are boxed in a dashed green line. The top 20% ATTO550 expressing cells were then bulk sorted on mean intensity and this population was lysed, quantified, and contrasted to the bulk sorted pool **(far right bottom panel)**.
(TIF)

S2 Fig The mutation spectrum induced by CRISPR and ZFN is observed at alternative loci. Two independent gRNAs or a ZFN pair for the OLFR613 locus were transfected CHO cells, the results from each modality were pooled and analyzed by TIDE. The binding sequence of the Zinc-finger proteins are showed in the bottom table.
(TIF)
