# Supplementary figures and images for: High throughput, efficacious gene editing & genome surveillance in Chinese hamster ovary cells
Source: PLoS One. 2019 Dec 19;14(12):e0218653. doi: 10.1371/journal.pone.0218653 (PMC6922373; doi:10.1371/journal.pone.0218653)

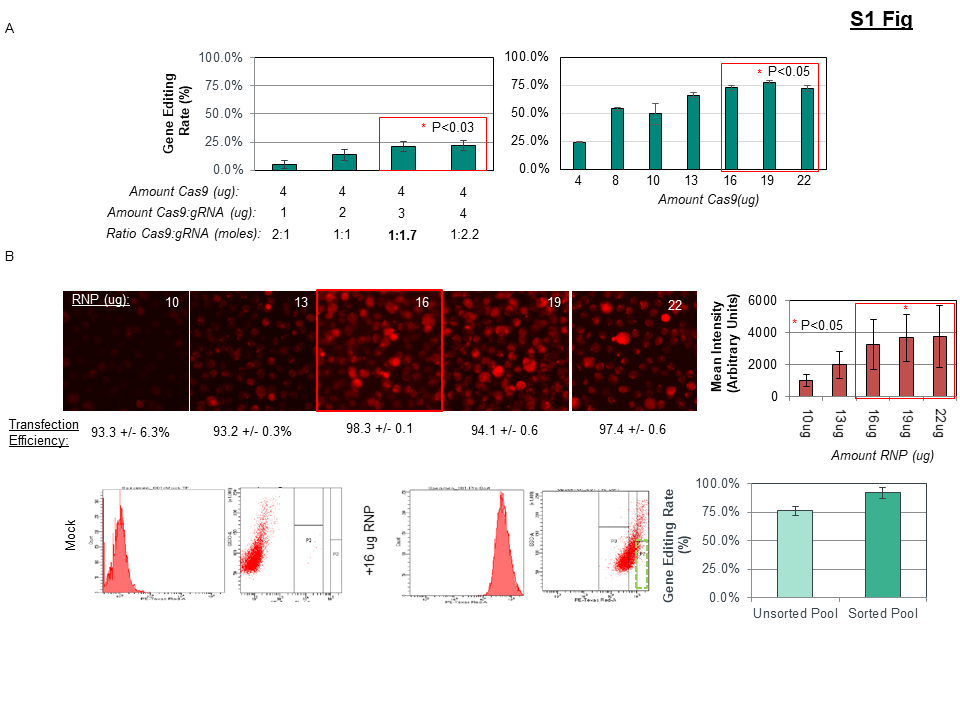

Supplement: S1 Fig — (A) CHO cells were transfected with 25 picomoles of Cas9 nuclease (4ug) while titrating the amount of gRNA (left hand panel). Cells were then transfected with the indicated amount of Cas9 nuclease while maintaining a fixed 4:3 nuclease:gRNA mass ratio (or 1:1:1.7 moles; right hand panel). The percent gene editing efficiency in each condition was then quantified and graphed. The red boxed bars in each graph demonstrates significance (p value indicated) vs other samples but not each other. (B) CHO cells were then transfected with the indicated amount of fluorescent RNP and imaged using fluorescent microscopy (center image panels). The mean fluorescent intensity of the cell population was quantified by microscopy (far right-hand panel) and percentage of transfected cells determined by flow-cytometry is demonstrated under each image. The representative fluorescent shift between the control and transfected cells, determined by flow cytometry is represented in the bottom hand panels and the top 20% expressers are boxed in a dashed green line. The top 20% ATTO550 expressing cells were then bulk sorted on mean intensity and this population was lysed, quantified, and contrasted to the bulk sorted pool (far right bottom panel). (TIF) [file pone.0218653.s001.TIF]

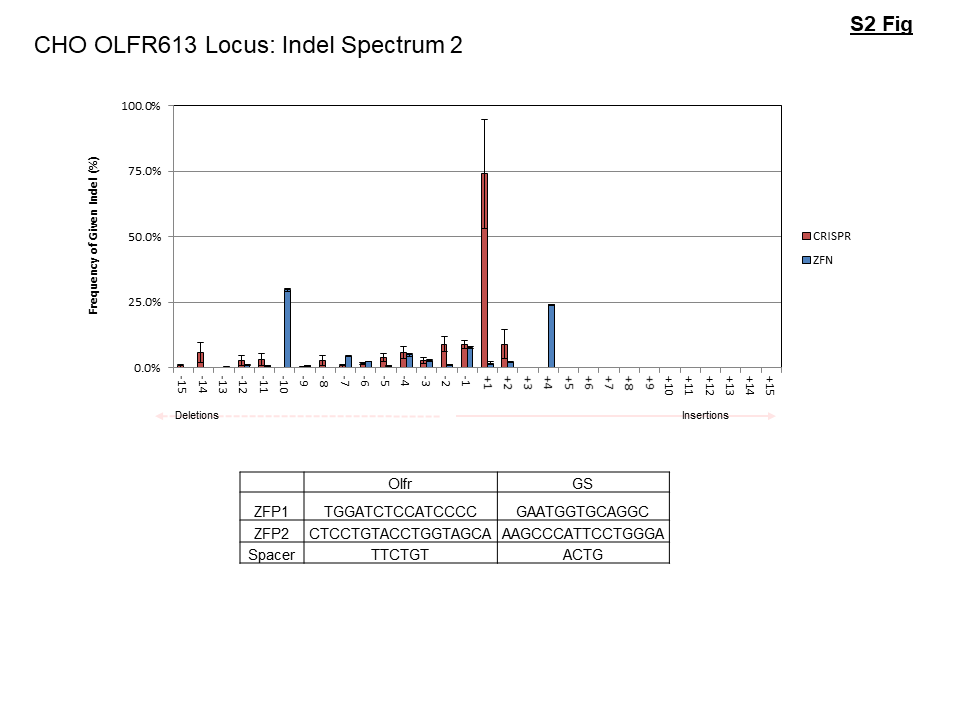

Supplement: S2 Fig — Two independent gRNAs or a ZFN pair for the OLFR613 locus were transfected CHO cells, the results from each modality were pooled and analyzed by TIDE. The binding sequence of the Zinc-finger proteins are showed in the bottom table. (TIF) [file pone.0218653.s002.TIF]
